# Evaluation and Treatment of Perioperative Corneal Abrasions

**DOI:** 10.1155/2014/901901

**Published:** 2014-02-04

**Authors:** Kira L. Segal, Peter M. Fleischut, Charles Kim, Ben Levine, Susan L. Faggiani, Samprit Banerjee, Farida Gadalla, Gary J. Lelli

**Affiliations:** ^1^Department of Ophthalmology, New York-Presbyterian Hospital, Weill Cornell Medical College, 1305 York Avenue, New York, NY 10065, USA; ^2^Department of Anesthesiology, New York-Presbyterian Hospital, Weill Cornell Medical College, Box 124, New York, NY 10065, USA; ^3^Division of Biostatistics & Epidemiology, Department of Public Health, Weill Cornell Medical College, New York, NY 10065, USA

## Abstract

*Purpose.* To evaluate perioperative risk factors for corneal abrasion (CA) and to determine current care for perioperative CA in a tertiary care setting. *Methods.* Hospital-based, cross-sectional study. In Operating Room and Post-Anesthesia Care Units patients, a comparison of cases and controls was evaluated to elucidate risk factors, time to treatment, and most common treatments prescribed for corneal abrasions. *Results.* 86 cases of corneal abrasion and 89 controls were identified from the 78,542 surgical procedures performed over 2 years. Statistically significant risk factors were age (*P* = 0.0037), general anesthesia (*P* < 0.001), greater average estimated blood loss (*P* < 0.001), eyes taped during surgery (*P* < 0.001), prone position (*P* < 0.001), trendelenburg position (*P* < 0.001), and supplemental oxygen en route to and in the Post-Anesthesia Care Units (*P* < 0.001). Average time to complaint was 129 minutes. 94% of cases had an inpatient ophthalmology consult, with an average time to consult of 164 minutes. The most common treatment was artificial tears alone (40%), followed by combination treatment of antibiotic ointment and artificial tears (35.3%). *Conclusions.* Trendelenburg positioning is a novel risk factor for CA. Diagnosis and treatment of perioperative corneal abrasions by an ophthalmologist typically require three hours in the tertiary care setting.

## 1. Introduction

Corneal abrasion (CA) is the most common ophthalmic injury in the perioperative period. Published data report an incidence range of 0.17%–44% [1–7]. While long-term complications of corneal abrasions are uncommon, the perioperative injury is unexpected, painful, and anxiety inducing for the patient. Patients may complain of blurry vision, tearing, redness, photophobia, and foreign body sensation in the eye [8]. In addition, discharge from the hospital can be delayed as the patient waits for an ophthalmology consultation before diagnosis can be made and treatment initiated.

Perioperative risk factors for CA have been reported in the literature and include increasing age of the patient, type of anesthesia received, length of surgery, and prone positioning [1, 2, 5, 6, 9–14]. Though cause of injury is often not identified, many abrasions are secondary to mechanical damage [1, 4, 15]. Various strategies, such as taping of eyelids and installation of paraffin based ointments into the conjunctival sac, have been suggested to decrease incidence.

Due to the self-regenerating nature of corneal epithelial cells, corneal abrasions generally resolve quickly with limited treatment. A combination of drops is the quickest and most comfortable way for the patient to achieve healing [16, 17]. Due to the simplicity of treatment options and extremely low risk for long-term damage, simple abrasions can be assessed and managed by nonspecialists. The Departments of Anesthesiology and Ophthalmology at New York-Presbyterian Hospital, Weill Cornell Medical College, initiated a study to identify new risk factors and to evaluate the time to diagnosis and treatment of corneal abrasions in the perioperative period. As a result of this study, a simple, standardized algorithm that begins immediately following initial report of symptoms or signs of perioperative eye injury has been developed.

## 2. Materials and Methods

An IRB-approved retrospective review of the medical records of all adult patients diagnosed with a perioperative CA over a two-year period (1/1/2007–12/31/2008) was conducted. All patients with a postoperative diagnosis of CA were included in the study; there were no patients excluded. Corneal abrasion was diagnosed using clinical history and exam finding consistent with fluorescein uptake of the corneal epithelium. A password protected CA database was created to record all patients with perioperative eye injury and patients in the control group. This database was compliant with HIPPA regulations.

A cohort of controls was selected randomly from all patients undergoing a surgical procedure from January 2007 to December 2008 who did not sustain corneal injury in the perioperative period. Selection of control cohort was accomplished by computer generated sample. Controls were intentionally, completely unmatched to allow for statistical assessment of a wide range of risk factors for abrasions. Therefore, the control cohort was not matched to the CA group in terms of age, type of surgery, day of surgery, or anesthesia team. Cross-analysis was performed when appropriate to further enhance the significance of comparisons.

The data were collected using an electronic medical record and downloaded into the CA and control databases, respectively. The investigators were blinded to all patient identifiers and to the identity of the attending anesthesiologist, resident anesthesiologist, and/or CRNA.

The patient records were analyzed for demographic data (gender, race, height, weight, and age). Intraoperative factors were recorded, including ASA status, surgical service, patient admission status, anesthesia type (general, monitored anesthesia care (MAC), local, or regional), operating room time, intubation type, patient position, eye protection, estimated blood loss, and whether CA occurred in the operating room. Factors analyzed in the PACU consisted of oxygen use, method of oxygen delivery, duration of oxygen usage, maximum oxygen flow, level of consciousness at the time of complaint, and whether CA occurred in the PACU or another unit. The perioperative care factors recorded included average time to complaint, completion of an ophthalmology consultation, average time to consultation, treatment recommendation, and long-term sequelae. Time to complaint was measured in minutes from the end of anesthesia administration to the first record of redness, tearing, difficulty opening eyes, or verbal complaint of ocular pain or foreign body sensation. Time to treatment was measured in minutes from the time of complaint to the time of prescribed ophthalmic medication. The precipitating factors analyzed included spectacle use, contact lens wear, history of dry eye syndrome, history of ocular surgery, other ocular history, allergy, and tobacco use.

A chi-square test of association or a Wilcoxon's rank sum test was used when appropriate. For contingency tables with low cell count, *P* values for the chi-square test were calculated based on Monte Carlo simulations (Hope, 1968). Cross-analysis on the groups was performed where appropriate. All statistical tests were performed using R 2.10.0 statistical software (R Development Core Team, 2009).

## 3. Results

Eighty six (0.11%) corneal abrasions occurred over the two-year interval examined, during which 78,542 procedures requiring anesthesia were performed. The CA group consisted of 57% males and 43% females as opposed to 42% males and 58% females in the control group (*P* = 0.0497, OR 1.854; 95% CI = 0.979−3.55). Race was not recorded for the majority of patients, but, for those patients in whom race was documented, Caucasian race accounted for 51% and 39% in the case and control groups, respectively. Average height and weight did not differ significantly between groups (Table 1). Average age was found to be significantly higher in the CA group (55 years) as compared to the control group (45 years), a finding consistent with prior studies (*P* = 0.0036).

Intraoperative risk factors were examined. The most common ASA status in both the control and CA groups was ASA 2. Urological surgery was the most common type of surgery in the CA group, and this differed significantly from the controls (31% versus 11%, *P* = 0.005). Robotic prostatectomy was the most common urological surgery performed (48%). Same day admission was more common in the CA group (66% versus 18%, *P* < 0.001). Operative times were longer for CA compared to control cases (3.85 hours versus 1.7, *P* < 0.001). Results from this study replicated prior findings as 95% of patients in the CA group versus 47% in the control group received general anesthesia (*P* < 0.001). Prone (6% versus 0%, *P* < 0.001) and trendelenburg positions (26% versus 6%, *P* < 0.001, OR = 5.721, 95% CI = 1.97–20.4) were found in a greater percentage of patients with CA. After controlling for trendelenburg position, urologic surgery was not significantly associated with CA (*P* = 0.11). A greater percentage of patients with CA had their eyes taped (94% versus 52%, *P* < 0.001). Average estimated blood loss was greater in the CA group (191 mL versus 90 mL, *P* < 0.001).

A greater percentage of patients with CA received supplemental oxygen en route to and in the PACU (69% versus 24%, *P* < 0.001). The main PACU, as opposed to the ambulatory PACU/other recovery sites, reported a greater percentage of corneal abrasions (66% versus 27%, *P* = 0.004). Oxygen type (nasal cannula versus facemask), duration of oxygen use, and maximum oxygen flow did not significantly differ between the two groups (Table 2).

Perioperative care factors were analyzed only for those patients with corneal abrasions. Average time to complaint was 129 minutes, with 94% of cases leading to completed ophthalmology consult. The time to consult was an average of 164 minutes (range = 0–1008 minutes, SD = 172 minutes) (Table 3). The most common treatment proposed by the consulting ophthalmologist was antibiotic ointment in combination with artificial tears (AT) (37.6%). There were no long-term ophthalmic sequelae (Table 4).

Other precipitating factors hypothesized to be risk factors for CA were analyzed and found not to differ significantly between groups. However, a trend toward CA for patients with previous ophthalmic history was identified. Glasses (64% versus 52%, *P* = 0.4206) and contact lens (12% versus 8%, *P* = 0.6157) were found to be more common in patients with CA. A greater percentage of patients with CA had a history of dry eye (14% versus 9%, *P* = 0.6334) and significant ocular history including glaucoma, cataracts, double vision, legally blind, lazy eye, or lid ectropion. Prior ophthalmologic surgery was more common in the control group (13% versus 8%, *P* = 0.2232). A history of allergy (medication, seasonal, food related, or material/other) was equally distributed between the two cohorts. Thirty-eight percent (38%) of the CA patients were smokers or had a history of smoking versus 32% of controls (*P* = 0.1949).

## 4. Discussion

Advanced age, general anesthesia, large estimated blood loss, same day admission (versus ambulatory), increased length of postoperative recovery in the PACU, and oxygen administration in the PACU are confirmed as posing greater risk for CA. Trendelenburg position has been newly identified as posing greater risk for CA. We found an increased incidence of CA with urologic surgery; however, when accounting for patient positioning, this was no longer significant. Paradoxically, this study found increased CA with taping of the eyelids. The rough application or removal of the tape and/or patient eye rubbing postoperatively may cause abrasion.

General anesthesia and advanced patient age are the most consistently cited risk factors in the literature. Oxygen use during transport/recovery and patient positioning are now emerging as influential risk factors. Cross and White, 1998, hypothesized that inadequate supply of oxygen to the cornea, lagophthalmos, decreased bells phenomenon, and decreased tear production during general anesthesia all contribute to corneal drying. During trendelenburg positioning, increased corneal thickness may occur as the result of elevated intravascular, episcleral venous and intraocular pressure. Longer and more complicated surgical procedures may lead to greater desiccation of the ocular surface by various mechanisms. Ultimately, comprised vitality of the corneal epithelial cells will increase propensity for sloughing and abrasion.

Although CA is not usually a sight threatening injury, it is a relatively common perioperative complication that causes immediate discomfort and concern for the patient. Additionally, it typically requires significant time and resource investment by both ophthalmology and anesthesiology teams. That anesthesiology consults ophthalmology prior to initiation of treatment necessitates greater waiting times for patients in pain and delays their disposition, resulting in higher medical costs for services rendered and decreased patient satisfaction. In this tertiary care hospital, findings show that symptoms of CA are recognized 129 minutes following termination of anesthesia. Patients wait another 164 minutes, on average, until an ophthalmologist can examine and diagnose the injury. When a diagnosis is made, treatment is most commonly a combination of antibiotic ointment with AT. Currently, followup with an ophthalmologist is only required if symptoms fail to improve within 24 hours. In this study, none of the patients was in need of continued treatment following discharge.

Based on these findings and the clinical experience of both ophthalmologists and anesthesiologists at New York-Presbyterian Hospital, an algorithm was formulated for the care of corneal abrasions in the perioperative period. This new protocol educates anesthesiologists to recognize the rudimentary signs and symptoms of CA and to initiate treatment immediately following an empiric diagnosis. In this algorithm, an ophthalmology consult is still requested for confirmation of the diagnosis, but initiation of treatment by the anesthesiology team affords patients the opportunity to leave the hospital prior to a consult. Patients who elect to leave the hospital are given an information sheet and instructed to seek followup with an ophthalmologist if symptoms fail to improve within 24 hours.

**Figure 1:**
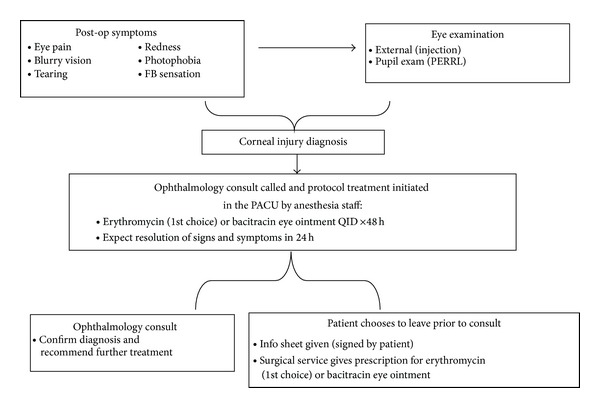
Corneal abrasion treatment algorithm.

This treatment algorithm suggests that, following corneal injury diagnosis, protocol treatment should be initiated in the PACU by anesthesiology staff. Treatment proposed includes either erythromycin (1st choice) or bacitracin ophthalmic ointment four times/day for 48 hours. This treatment has minimal risk for side effects, the most common being a contact-type allergic reaction. Although NSAIDs have been shown to improve pain, this treatment option was purposely left out of the algorithm for two reasons. The first is for simplicity of the protocol. Since anesthesiology staff will be treating patients with eye injury, the algorithm should be easy to follow and to reproduce on a larger scale. In addition, if patients elect to leave the PACU prior to an ophthalmology consult, pain is the most likely symptom that will bring them to see an ophthalmologist if they fail to improve within 24 hours. If patients choose to wait for a consult, the ophthalmologist may discuss the risks and benefits of adding a topical NSAID (Figure 1).

This treatment algorithm has been launched at the New York-Presbyterian Hospital's Weill Cornell campus with collaboration between the Departments of Anesthesiology and Ophthalmology. To establish this protocol, the ophthalmologist spent time training the anesthesiologist staff in the recognition of signs and symptoms of CA and the proper administration of eye ointment. The expected outcome for this new protocol will be the improved management of CA with faster time to treatment for patients, higher patient satisfaction, and decreased utilization of unnecessary medical resources. Additional studies of the safety, efficacy, and reproducibility of this protocol are warranted.

Limitations include the retrospective nature of this study. Perioperative abrasions are, by definition, diagnosed at completion of surgery. Given the relatively small numbers with CA yearly compared to number of surgeries performed, powering a prospective study would be difficult. As a result of the retrospective design, intraoperative factors could not readily be examined. Bias could be introduced with an unmatched cohort. Despite this, the unmatched design was intentionally selected to allow for examination of a wide range of potential risk factors. Further, cross-analysis was performed to enhance significance of comparisons.

Trendelenburg position is newly identified as a risk factor for corneal abrasion, which occurs in 0.11% of all surgical procedures. The authors recommend a treatment algorithm in conjunction with anesthesia PACU staff to expedite treatment of perioperative abrasions.

## Figures and Tables

**Table 1:** Patient demographics.

	Corneal abrasion group	Control group	*P* value
*N*	86	89	
Average age	55 (13–86)	45 (1–83)	0.0036
Gender (% male)	57	37	0.05
Average height (inches)	67 (50–86)	65 (27–118)	
Weight (kg)	78 (50–86)	67 (10–176)	

**Table 2:** Statistically significant risk factors.

	Corneal abrasion group	Control group	*P* value
Urological surgery (*N*)	27	10	<0.001
Same day admission (*N*)	57	16	<0.001
General anesthesia (*N*)	82	42	<0.001
Prone position (*N*)	5	0	<0.001
Trendelenburg position (*N*)	22	5	0.0028
Eyes taped during surgery (*N*)	81	46	<0.001
Estimated blood loss (mL)	191	90	<0.001
Main PACU recovery (*N*)	57	33	0.0045
Oxygen use (transport/in PACU) (*N*)	59	21	<0.001

**Table 3:** Perioperative care factors.

Average time to complaint (minutes)	129 (−15–515)
Followup with ophthalmology (% yes)	94%
Average time to consult (minutes)	164 (0–1008)
Long-term sequelae	0%

**Table 4:** Treatment for corneal abrasion.

Treatment	%
AT only	40%
Antibiotic only	10.6%
Bacitracin	2.4%
Erythromycin	7%
Polytrim ophthalmic	1.2%
Antibiotic and AT	35.3%
Bacitracin	18.8%
Erythromycin	12.9%
Polytrim ophthalmic	2.4%
Moxifloxacin	1.2%
Two antibiotics and AT	9.4%
Moxifloxacin + erythromycin	2.4%
Erythromycin + polytrim	3.5%
Bacitracin + polytrim	2.4%
Bacitracin + moxifloxacin	1.2%
Two antibiotics (erythromycin + polytrim)	1.2%
Lubricant	1.2%
One antibiotic and cycloplegic	1.2%
Two antibiotics and cycloplegic (bacitracin + moxifloxacin)	1.2%
